# J-bone graft with double locking plate: a symphony of mechanics and biology for atrophic distal femoral non-union with bone defect

**DOI:** 10.1186/s13018-020-01636-3

**Published:** 2020-04-15

**Authors:** Jian Lu, Shang-Chun Guo, Qi-Yang Wang, Jia-Gen Sheng, Shi-Cong Tao

**Affiliations:** 1 Department of Orthopaedic Surgery, Shanghai Jiao Tong University Affiliated Sixth People’s Hospital, 600 Yishan Road, Shanghai, 200233 China; 2 Institute of Microsurgery on Extremities, Shanghai Jiao Tong University Affiliated Sixth People’s Hospital, 600 Yishan Road, Shanghai, 200233 China; 3 The Third Affiliated Hospital of Soochow University, Changzhou, 213003 Jiangsu China

**Keywords:** Distal femur non-union with bone defect, J-shaped iliac crest bone graft, Finite element analysis

## Abstract

**Objective:**

Atrophic distal femur non-union with bone defect (ADFNBD) has been a worldwide challenge to treat due to the associated biological and mechanical problems. The purpose of this study was to introduce a new solution involving the use of a J-shaped iliac crest bone graft (J-bone) combined with double-plate (DP) in the treatment of femoral non-union.

**Methods:**

Clinically, 18 patients with ADFNBD were included in this retrospective study and were treated with a combination of J-bone graft and DP. The average follow-up time was 22.1 ± 5.5 months (range, 14 to 34 months). The imaging information and knee joint activity tests and scores were used to evaluate the time to weight-bearing, the time to non-union healing, and the knee joint mobility. A finite element analysis was used to evaluate the differences between the following: (1) the use of a lateral locking plate (LLP) only group (LLP-only), (2) a DP only group (DP-only), (3) a DP with a J-bone group (DP+J-bone), and (4) an LLP with a J-bone group (LLP+J-bone) in the treatment of ADFNBD. A finite element analysis ABAQUS 6.14 (Dassault systems, USA) was used to simulate the von Mises stress distribution and model displacement of the plate during standing and normal walking.

**Result:**

All patients with non-union and bone defect in the distal femur achieved bone healing at an average of 22.1 ± 5.5 months (range, 14 to 34 months) postoperatively. The average healing time was 6.72 ± 2.80 months. The knee Lysholm score was significantly improved compared with that before surgery. Under both 750 N and 1800 N axial stress, the maximum stress with the DP+J-bone structure was less than that of the LLP+J-bone and DP-only structures, and the maximum stress of J-bone in the DP+J-bone was significantly less than that of the LLP+J-bone+on structure. The fracture displacement of the DP+J-bone structure was also smaller than that of the LLP+J-bone and DP-only structures.

**Conclusion:**

J-bone combined with DP resulted in less maximum stress and less displacement than did a J-bone combined with an LLP or a DP-only graft for the treatment of ADFNBD. This procedure was associated with less surgical trauma, early rehabilitation exercise after surgery, a high bone healing rate, and a satisfactory rate of functional recovery. Therefore, a combination of J-bone and DP is an effective and important choice for the treatment of ADFNBD.

## Introduction

Distal femoral fracture is a rare type of fracture with a reported incidence of 8.7/100,000/year in 2005–2010 [1]. These fractures are caused by high-energy damage in young people or low-energy damage in the elderly with concomitant osteoporosis. Retrospective studies have reported that the complication rates of malunion, non-union, infection, or death after distal femoral fracture are as high as 15–20% [2–5]. Due to the anatomical shape of the femur [6] and the mechanical effect of the distal femur, atrophic distal femoral non-union with bone defect (ADFNBD) is one of the most difficult subtypes of distal femoral non-union [7] and varus deformity with medial posterior bone defect [8]. Poor bone mass and bone defects at the distal femur present a huge challenge for orthopedic surgeons around the world.

The greatest challenge in the treatment of ADFNBD is how to provide sufficient stability and potential for osteogenesis. A J-shaped iliac crest is a bicortical structural bone graft [9–11] used in the conventional surgical procedure for reconstructing the articular hernia to resolve shoulder instability.

The purpose of this study was to demonstrate a possible new method called double-plate with J-bone for treating ADFNBD. In theory, the J-bone provides superior autologous bone osteogenesis, and its bicortical structure can overcome the lack of stability of the medial posterior side. Therefore, a main lateral locking plate (LLP), a protective medial locking plate (MLP), and a supporting J-bone can be applied for treating ADFNBD.

In this study, multiple groups of models were constructed using finite element analysis, and the maximum stress and changes in fracture clearance of the internal fixation and J-bone were compared under simulated standing and normal walking conditions. Preoperative and postoperative radiographic data and knee joint evaluation scales were also used to evaluate the effectiveness of a steel double-plate (DP) combined with a J-bone for the treatment of ADFNBD.

## Methods

### Clinical investigation

#### Patients and methods

This study protocol was approved by the independent ethics committee of the Shanghai Sixth People’s Hospital Review Committee. All patients provided informed consent. The patient inclusion criteria were as follows: (1) non-union, defined as failure of fracture healing over at least 9 months or no evidence of progressive healing over three consecutive months [12], and imaging evidence supporting the diagnosis of atrophic non-union; (2) voluntarily choosing to use a double-plate with a J-bone; (3) voluntary participation in clinical trials; and (4) signing of an “informed consent for operation” form. Eighteen patients treated between Jan 2017 and Sept 2018 who met the inclusion criteria were enrolled. The details of these patients are shown in Table 1.

**Table 1:** Details of patients treated with a J-bone combined with a steel double-plate for distal femoral non-union with a bone defect

Patient number	Age (years)	Sex	Mechanism of injury	Interval between non-union (months)	Previous treatment	Time to union (months)	Complication	Follow-up (months)
1	41	F	MVA	20	LLP	6	None	34
2	54	M	MVA	12	LLP	8	SWI	29
3	49	M	MVA	24	LLP	6	None	28
4	28	M	MVA	10	LLP	8	None	27
5	62	M	FALL	5	LLP	6	None	27
6	59	M	MVA	4	LLP	3	None	26
7	35	F	MVA	24	LLP	12	None	25
8	42	F	MVA	13	LLP	3	None	22
9	48	F	MVA	48	LLP	6	None	20
10	48	M	Injury	9	LLP	6	Knee stiffness	20
11	63	F	MVA	7	LLP	6	SWI	20
12	53	F	MVA	12	LLP	9	None	20
13	49	F	MVA	9	LLP	12	None	19
14	35	M	Injury	10	LLP	9	None	19
15	47	M	MVA	9	LLP	3	None	18
16	60	F	MVA	6	LLP	3	None	16
17	39	F	MVA	10	LLP	9	None	14
18	47	M	MVA	48	LLP	6	None	14

*LLP* lateral locking plate, *F* female, *M* male, *MVA* motor vehicle accident, *SWI* superficial wound infection

#### Surgical technique

The four surgical methods are all in under general anesthesia; all patients were placed in a supine position. First, the bicortical iliac crest bone graft was harvested and prepared as previously described [13, 14]. After molding of the bicortical iliac crest graft with an oscillating saw and a high-speed burr in a J-shaped fashion, the outside of the femur was exposed along the previous outer lateral incision on the thigh and the original internal fixation was removed to further expose the non-union of bone. Next, the scar tissue, original callus, sclerotic bone, and dead bone were removed, and the medullary cavity of the broken ends was opened on both sides. After adjusting the limb length, rotation, and angulation of the fracture, it was stabilized temporarily using a K-wire, and a long LLP was subsequently placed outside the fractured femur. Next, a minimally invasive longitudinal incision (3–7 cm) was made on the medial side of the non-union site, followed by deep dissection to expose the non-union. The keel of the J-bone graft was impacted on the graft with a mallet into the medial defect to obtain press-fit fixation. Finally, the medial locking compression plate (LCP) (3.5-mm hole) was fixed to the medial side of the distal femur for protection and support (Fig. 5). The drainage tube was routinely placed for no more than 72 h after surgery to prevent postoperative hematoma. All patients were treated with antibiotics to prevent infection.

The other three surgical methods also used the original longitudinal incision, and all removed the original internal fixation and clear scar tissue and dead bones. In the LLP-only group, a long LCP was fixed on the outside of the fracture femur. On the basic of LLP-only group, the LP+J-bone group also tapped the trimmed J-shaped iliac crest bone graft (J-bone) as described earlier on the medial side of distal femur after removed scar tissue and dead bone. Different from LP+J-bone, the DP group added a 3.5-mm medial LCP which was fixed on the medial side of the distal femur through a medial incision. Cancellous bone pieces are employed, in all groups, to promote bone healing in defects.

#### Postoperative management

All patients were advised to perform active functional exercises for the quadriceps on the first postoperative day. Active function of the knee joint was assessed 2 weeks after surgery. Partial weight-bearing was permitted 4 weeks after surgery based on the clinical and imaging evidence of fracture healing.

The patients were evaluated using imaging data (CT scan) and functional tests (Lysholm Knee Scoring Scale scores [15]) 1, 3, 6, and 12 months after surgery. Bone healing was defined as the absence of bone or weightless pain in the non-union site and the presence of three cortical bridges on the anterior and lateral imaging data.

### Finite element analysis

#### Three-dimensional models

Using enhanced computed tomography (CT) with slicing of 0.625 mm at a 20-cm slice distance, the femur of a healthy young male (age 20 years, height 178 cm, body weight 85 kg) was scanned to obtain raw imaging data of a normal femur. Informed consent was obtained from the subject to use his radiologic data for research purposes. The images, in Digital Imaging and Communications in Medicine (DICOM) format, were imported into Mimics 20.0 (The Materialise Group, Leuven, Belgium) in order to generate a three-dimensional (3D) model. Bone mineral density parameters were calculated according to the following equation by Reina-Romo et al. [16] based on CT data:

1 $$ \rho =0.000968\times \mathrm{HU}+0.5,\left(\mathrm{g}/{\mathrm{cm}}^3\right) $$

2 $$ E=\left\{\begin{array}{c}2014{\rho}^{2.5},\left(\mathrm{MPa}\right)\kern1.75em \rho\ \mathrm{no}\ \mathrm{more}\ \mathrm{than}\ 1.2\mathrm{g}/{\mathrm{cm}}^3\ \\ {}1763{\rho}^{3.2},\left(\mathrm{MPa}\right)\kern1.75em \rho\ \mathrm{granter}\ \mathrm{than}\ 1.2\mathrm{g}/{\mathrm{cm}}^3\end{array}\right. $$

3 $$ \upnu =\left\{\begin{array}{c}0.20,\kern1.25em \rho\ \mathrm{no}\ \mathrm{more}\ \mathrm{than}\ 1.2\mathrm{g}/{\mathrm{cm}}^3\\ {}0.32,\kern1.25em \rho\ \mathrm{granter}\ \mathrm{than}\ 1.2\mathrm{g}/{\mathrm{cm}}^3\end{array}\right. $$

The Geomagic software was used to sample and build the geometry and surface, and the resulting basic 3D model was compiled and meshed in HyperMesh 14.0 (Altair Engineering Inc., Troy, MI, USA). In 3-Matic 11.0 (Materialise, Leuven, Belgium), a 15-mm transverse osteotomy plane of 6.5 cm was made near the joint line to simulate distal femur mechanical structure instability with bone defect [17, 18].

**Fig. 1:**
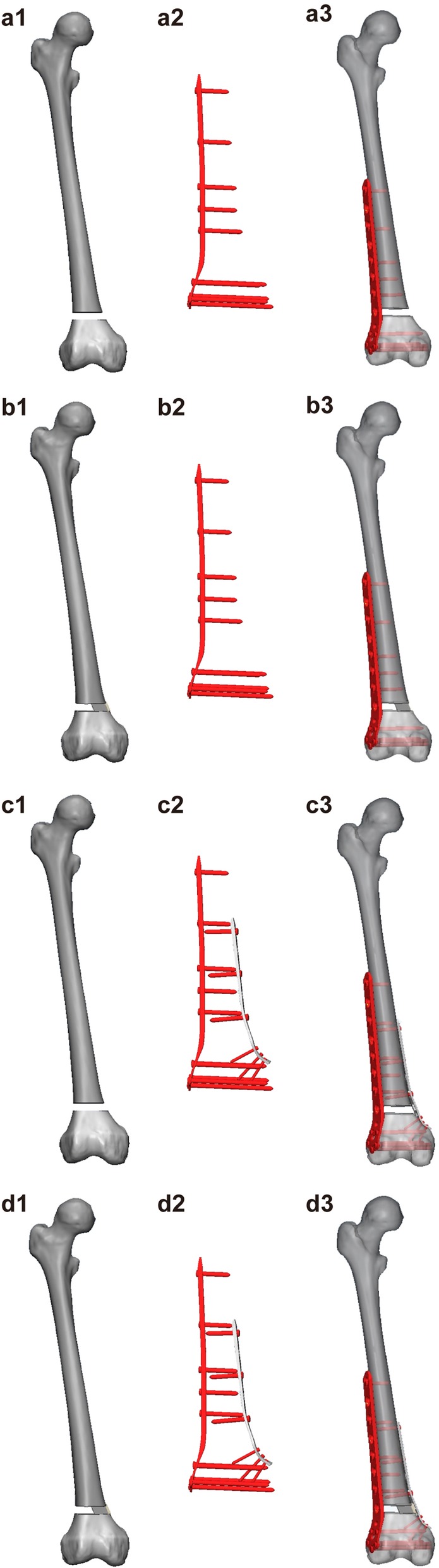
Construction of the four three-dimensional models. a1–a3: model 1 is built with only a lateral locking plate (LLP-only); b1–b3: model 2 is built with a J-bone+LLP; c1–c3: model 3 is built with only a double locking plate (DP-only); d1–d3: model 4 is built with J-bone+DP. *LLP* lateral locking plate, *DP* double-plate

According to the blueprint provided by the manufacturer, the LLP, MP, and screws (Synthes, 3.5-mm LCP) were designed and modified using Solid Works 14.0 (Dassault Systèmes Solid Works Corp., Concord, MA, USA). The geometric parameters of plates and screws were loaded into 3-Matic 11.0 (Materialise, Leuven, Belgium), and four case models were established according to the experiment (Fig. 1). The threaded surface of the screw was replaced by a smooth surface, and the size of the surface corresponded to the screw data provided by the manufacturer [19]. Finally, HyperMesh 14.0 (Altair Engineering Inc., Troy, MI, USA) was used to mesh these combined models into 1 mm, and a finite element model was developed for mechanical analysis with ABAQUS 6.14 (Dassault Systèmes Solid Works Corp., Concord, MA, USA).

#### Material properties and boundary conditions

We assumed that both the femur and the strut were linear, isotropic, and elastic [20]. Cortical and trabecular bones were assigned a Young’s modulus (E) of 16.7 GPa and 0.155 GPa, respectively, and the Poisson’s ratios were 0.26 and 0.3 [17, 21], respectively (Table 2). The less invasive stabilization system (LISS) plate, medial plate, and screws were made of a titanium alloy (Ti-6AL-4V) (Table 2). According to early scholars [22, 23], the interface between the plate and the screws was modeled using a surface-surface contact element that allows separation and sliding. Considering that the internal fixation was locked, the rigid connection between the screws and the plate holes was simulated [24]. The friction coefficient of bone and bone interaction was 0.46, and that of bone and implant interaction was 0.3 [25]. In order to prevent rigid motion during the analysis, the femoral head was limited to a plane perpendicular to the loading vector, and the distal femur was fixed in all degrees of freedom [26].

**Table 2:** Material properties used for each type of structure

Components	Ti-6AL-4V	Bone	
		Cortical	Trabecular
Young’s modulus (GPa)	105	16.7	0.155
Poisson’s ratio	0.35	0.26	0.3

**Fig. 2:**
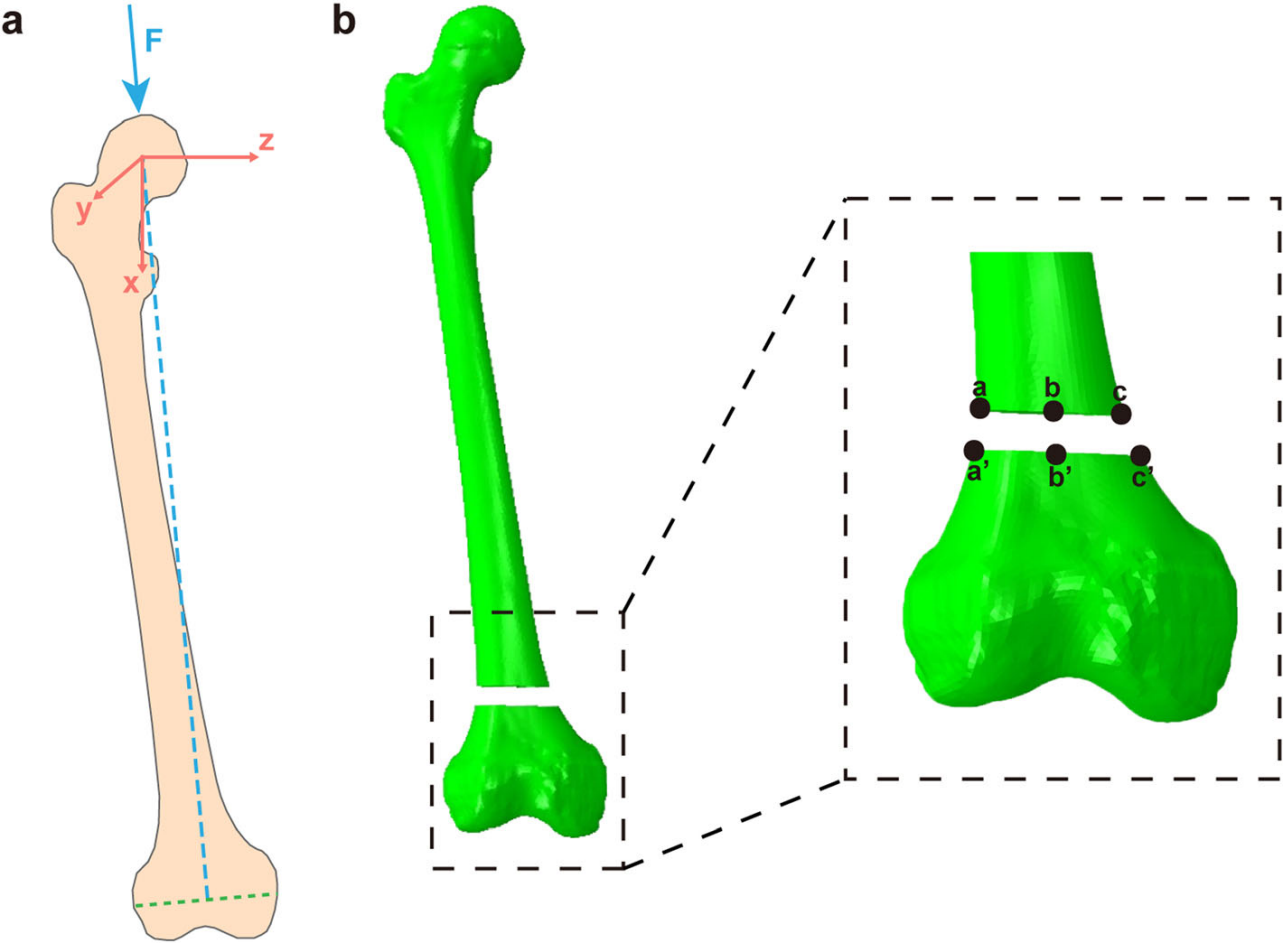
Calculations of loading force and fracture gap. **a** Schematic of the loading force from the focal point of the femoral head to the midpoint of the femoral condyle. **b** Three lines from a to a’, from b to b’, and from c to cc’ are used to calculate the axial micromotion of the fracture gap

In this study, all models were tested with 750 N (one-leg standing load force of 100% of the body weight [27]) and 1800 N (normal walking load force 238% body weight [28]) of adduction loads applied. The lateral displacement (aa’), middle displacement (bb’), and medial displacement (cc’) at the non-union gap were calculated and the change in height was observed (Fig. 2).

#### FEA

ABAQUS 6.14 (Dassault Systèmes, Solid Works Corp., Concord, MA, USA) was used to obtain the von Mises stress distribution of the LCP and J-bone during simulated standing and normal walking. Axial fretting was measured at the end gap to detect the stability of the model.

#### Statistical analyses

SPSS statistical software package 24.0 (SPSS Inc., Chicago, IL, USA) was used to analyze the relevant data. The chi-square test was used to compare the preoperative and postoperative “excellent” and “good” scores, and *P* < 0.05 was considered statistically significant.

## Results

### Results of the clinical study

Eighteen patients (nine females and nine males, mean age 47.7 years, range 28–62 years) were operated on using a DP with a J-bone. All 18 patients achieved primary bone healing, and the mean time to weight-bearing walking was 5.5 months (range 3–12 months) after surgery. At the last follow-up, no patients had developed postoperative limb malformations (more than 5° in the coronal, sagittal, or rotational deformities, or more than 1 cm in leg length). The rate of “excellent” and “good” Lysholm Knee Scoring Scale scores improved from 0% before surgery to 94.44% at 3 months after surgery (Table 3). Three patients had surgery-related complications. Two of them developed a superficial wound infection, which was normalized by irrigation and debridement. There was also one case of knee stiffness. After the non-union was healed, the function of the knee joint was improved by removing the internal fixation and releasing the quadriceps.

**Table 3:** Comparison of Lysholm Knee Scoring Scale scores before and after surgery

	*N*	Lysholm Knee Scoring Scale				Excellent and good rate
		Excellent	Good	Fair	Poor	
Preoperative	18	0 (0.00)	0 (0.00)	10 (55.56)	8 (44.44)	0 (0.00) *
Postoperative (3 months)	18	2 (11.11)	15 (83.33)	1 (5.56)	0 (0.00)	17 (94.44)

Data are presented as number (percentage)

*P < 0.05, preoperative vs. postoperative at 3 months

Figure 5 shows imaging data of patient 1 from the time of trauma to the time of treatment and follow-up.

### Results of the FEA

**Fig. 3:**
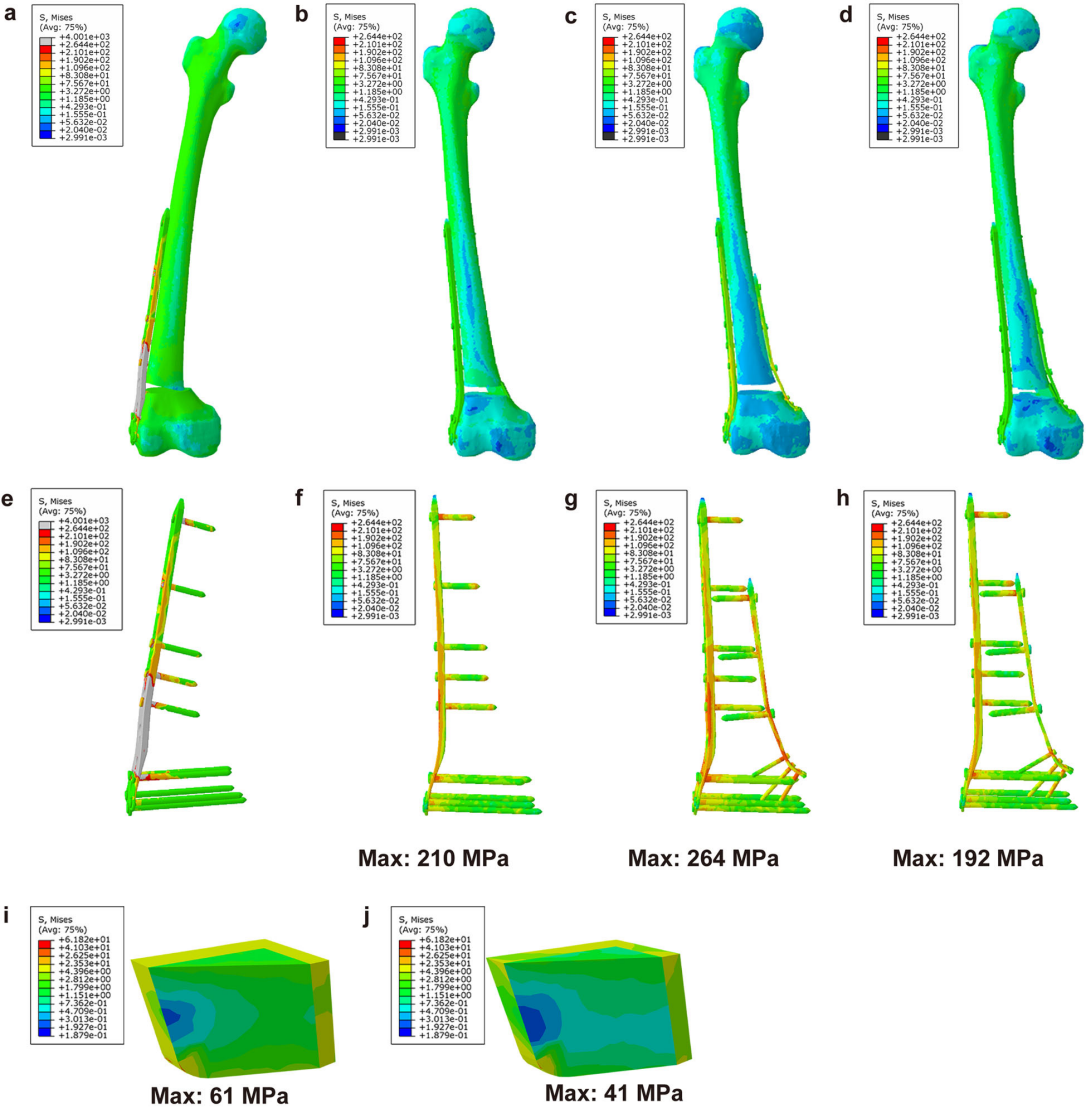
Overall von Mises stress (VMS) distribution in each model under 750 N axial stress. **a** Overall stress with LLP-only. **b** Overall stress with LLP+J-bone. **c** Overall stress with DP-only. **d** Overall stress with DP+J-bone. **e** Plate stress with LLP-only. **f** Plate stress with LLP+J-bone. **g** Plate stress with DP-only. **h** Plate stress with DP+J-bone. **i** J-bone stress with LLP+J-bone. **j** J-bone stress with DP+J-bone. *LLP* lateral locking plate, *DP* double-plate

**Fig. 4:**
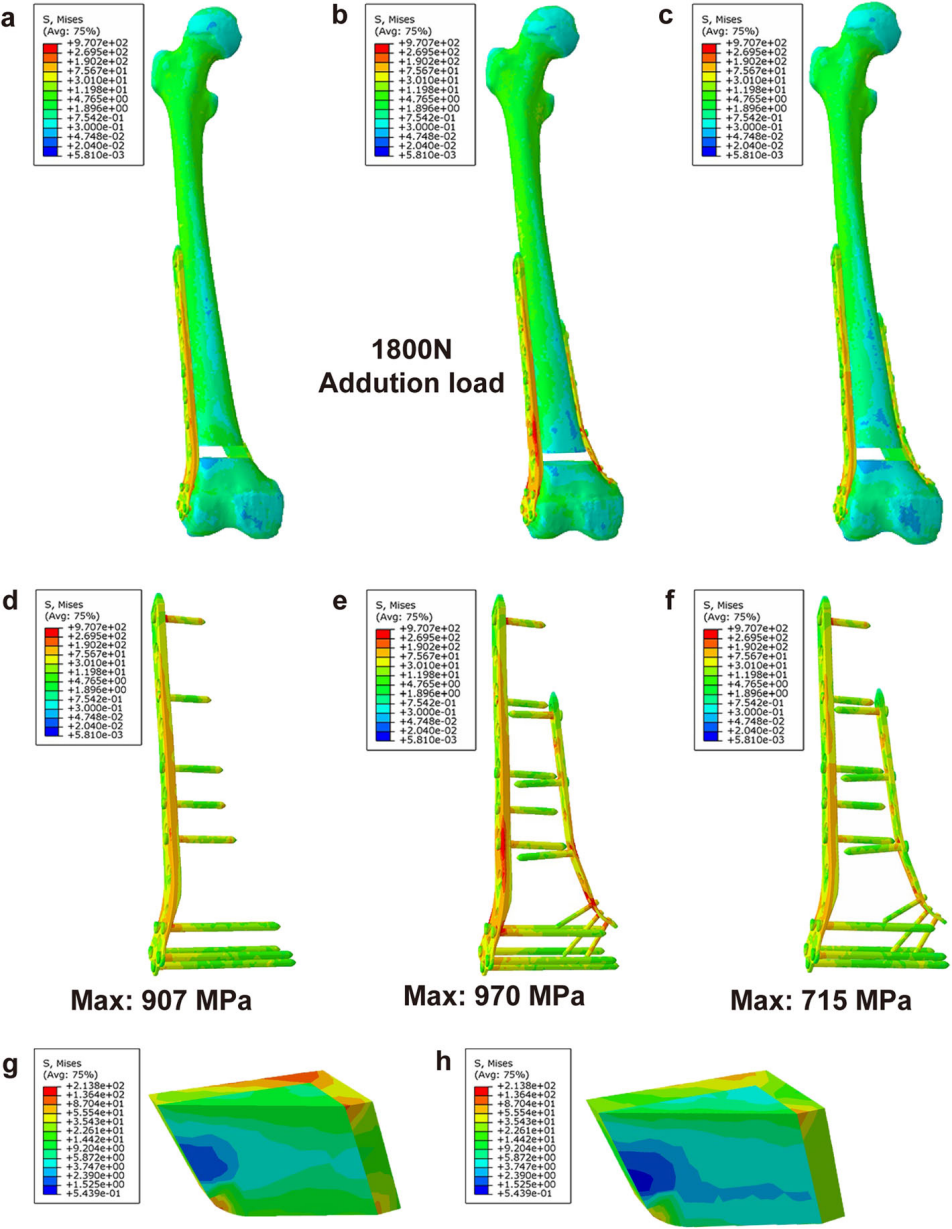
Von Mises stress (VMS) distribution in each model under 1800 N axial stress. **a** Overall stress with LLP+J-bone. **b** Overall stress with DP-only. **c** Overall stress with DP+J-bone. **d** Plate stress with LLP+J-bone. **e** Plate stress with DP-only. **f** Plate stress with DP+J-bone. **g** J-bone stress with LLP+J-bone. **h** J-bone stress with DP+J-bone. *LLP* lateral locking plate, *DP* double-plate

The numerical values of the stress distribution of each treatment group are shown in Table 4. Under 750 N of axial force, higher stress was found with the LLP-only graft and may have led to a higher risk of failure; however, the specific value could not be calculated. Maximum stress was placed on the J-bone in DP+J-bone and LLP+J-bone graft structures on the cortical bone on the medial and posterior sides of the distal femur, and the J-bone of the DP with a J-bone graft was significantly smaller than that of the LLP with a J-bone. Furthermore, under the axial stress of 750 N, the plate for the DP-only structure was under 1.38 times and 1.26 times as much stress as the DP+J-bone and LLP+J-bone structures, respectively (Fig. 3). Under 1800 N stress, the mean stresses around the plate for the DP-only structure were 1.36 times greater than that of the DP+J-bone group and 1.07 times greater than that of the LLP+J-bone group (Fig. 4).

**Table 4:** Maximum (mean) values of stress on the plate and J-bone in the three models under two levels of stress loading

Adduction load (N)	DP+J-bone		LLP+J-bone		DP-only
	Plate (MPa)	J-bone (MPa)	Plate (MPa)	J-bone (MPa)	Plate (MPa)
750	192	41	210	61	264
1800	715	134	907	213	970

*DP* double-plate, *LLP* lateral locking plate

The model displacement values for different loads in the three implant groups are shown in Table 5.

**Table 5:** Displacement values of the non-union area of the three models under two levels of stress loading

Internal fixation	Adduction load (*N*)	Displacement (mm)		
		aa’	bb’	cc’
DP+J-bone	750	0.052	0.052	0.037
	1,800	0.191	0.213	0.118
LLP+J-bone	750	0.059	0.059	0.053
	1800	0.228	0.216	0.162
DP-only	750	0.184	0.185	0.214
	1800	0.766	0.757	0.840

*DP* double-plate, *LLP* lateral locking plate, *aa*’ lateral displacement, *bb*’ middle displacement, *cc*’ medial displacement

## Discussion

**Fig. 5:**
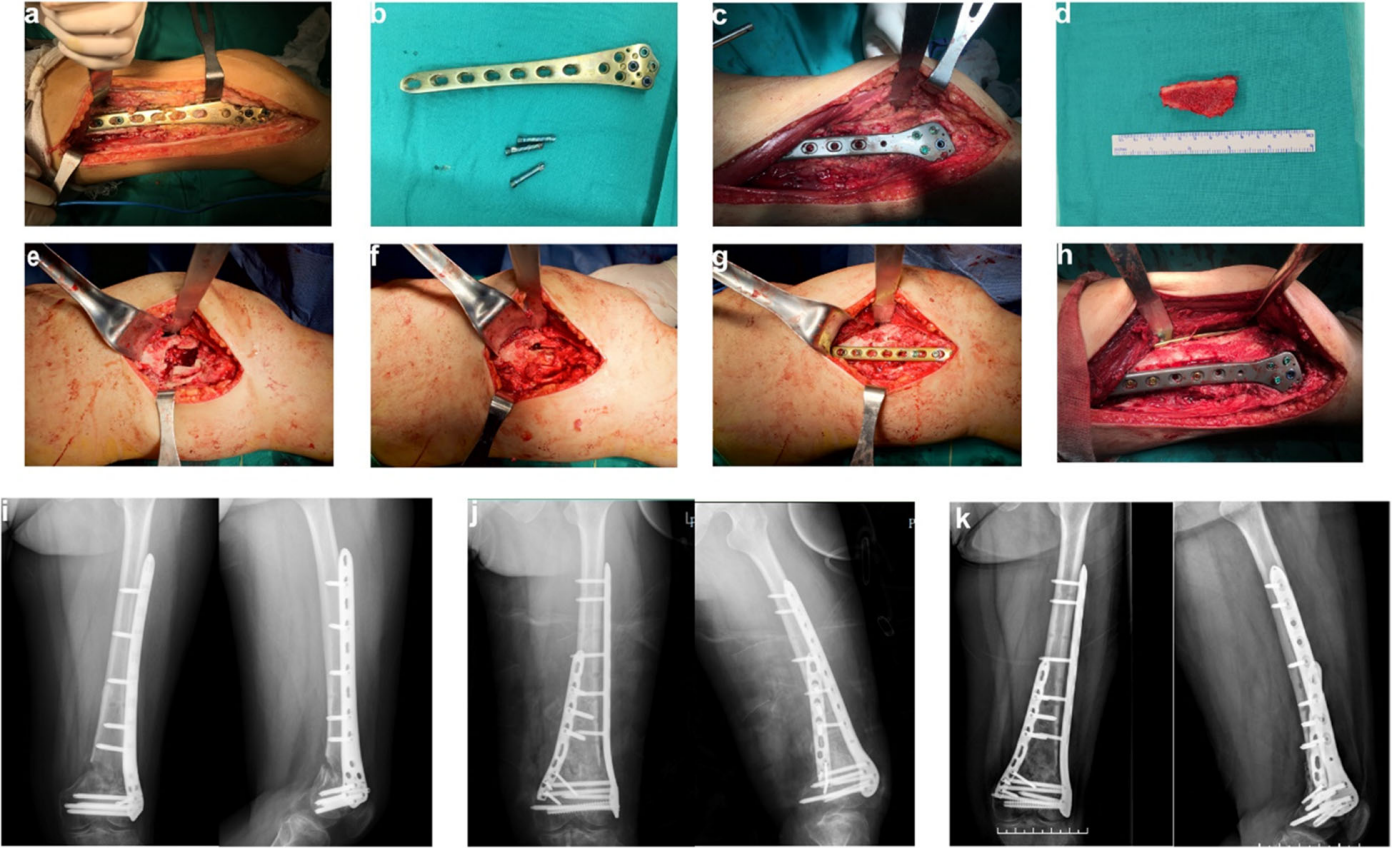
Surgical procedure and radiographic findings of a 39-year-old female patient. **a** The original internal fixation device is exposed. **b** The original internal fixation device is removed. **c** The lateral plate is fixed on the outside of the femur. **d** Image shows the J-bone graft before press-fit insertion. **e** Image shows the fenestration in the non-union of medial distal femur. **f** Image shows the tap J-bone graft on the medial side of the distal femur. **g** Image shows the medial plate protection J-bone. **h** The DP is grafted with J-bone. **i** Non-union is seen 10 months following surgery. **j** The plate is exchanged and grafted with a J-bone. **k** Three months following grafting, union is achieved. *DP* double-plate

Non-infectious distal femoral non-union can be divided into hypertrophic non-union and atrophic non-union according to the imaging data. Hypertrophic non-union requires replacement with a stronger fixing device due to a lack of good mechanical fixation. The fracture end of atrophic non-union lacks callus and cartilage due to lack of cells and blood supply, leading to proliferation and hardening of large fractures and atrophy of the fracture ends; therefore, the fracture site may be sclerotic or osteopenic [29]. Additionally, the lack of bone supports on the medial and posterior sides of the distal femur can cause instability, leading to varus tendencies or rotation [6]. The anatomy of the distal femur is complex, and the fracture area is accompanied by obvious scar tissue and poor blood flow at the stump, which presents great challenges in clinical treatment. For ADFNBD, successful treatment requires restoration of a painless, well-aligned knee with a satisfactory range of motion that maintains good alignment of the entire lower limb. Instability is the main cause of non-union; thus, it is better to fix it using a stable method and reduce the blood supply as little as possible [30]. A lack of bone morphogenetic cells or blood supply is also an important cause of non-union with bone defect [31] (Fig. 5).

Although there are many treatments for non-union of the distal femur, there is still no uniform standard. Previous reports have described the results of a variety of techniques for the treatment of distal femoral non-union. Intramedullary nails can minimize soft tissue injury [32, 33], but there is a risk of malunion in this correction, as well as obvious shortening of the non-union in the dynamic compression [34, 35]. The llizarov technique has the advantage of restoring the anatomical location of the non-union and increasing the stability of bone healing, leading to early weight-bearing. However, this technique is associated with a high probability of complications such as infection and the need for multiple operations [36, 37]. LCPs are favored due to their minimal trauma, small periosteal damage, and high stability [38]. Double locking plates provide continuous non-union site compression and the chance to clear dead bones. Maimaitiyiming et al. reported successful cure in 14 out of 37 patients with femoral fracture non-union using a double locking plate combined with bone grafting. Consequently, exposure of the non-union site, removal of the failed previous hardware, removal of fibrous scar tissue, correction of deformities, full bone grafting, and stable mechanical fixation are strategies for ADFNBD treatment. Jiang et al. [39] proved that the application of double locking plates with a fibular autograft is a promising method for diaphyseal femur fracture non-union; however, due to the possible complications of obtaining a graft from the fibular, it is only recommended for severe non-union. Treatment with DP combined with bicortical iliac bone provides a mechanical and biological environment, and unlike fibular grafts, it is not associated with serious complications; therefore, it may be a suitable alternative treatment.

In this study, we introduced a new method for treating ADFNBD. A total of 18 patients with non-union of the distal femur were included in this retrospective study, and these patients were treated with double-plates. The average follow-up time was 12 months. No cases of bone non-union or internal fixation rupture occurred. Before surgery, due to the defect of the distal femur, the affected limb was not able to bear weight or engage in exercise, resulting in poor knee joint mobility. One month after the operation, the healing status was judged based on imaging data and physical examination, and guided functional exercises were started. Early recovery after surgery and knee movement satisfaction rates were higher, and the knee joint mobility in this group was significantly improved compared to that before surgery. In order to further evaluate the performance of the new configuration, we conducted a finite element analysis, and the results showed that, compared with the use of an LLP+J-bone and a DP-only, the use of a DP+J-bone showed excellent stability in both simulated standing and walking conditions. Walking is the most common exercise method for patients with lower limb fractures [40]. However, in the case of simulated walking, the maximum stress of the LCP in the DP-only (970 MPa) and the LLP+J-bone (907 MPa) groups exceeded the yield strength of the Ti-6Al-4V alloy (889–921 MPa) [41]. Moreover, the displacement results indicated that the DP+J-bone provided higher stability than did the other two groups under 1800 N axial stress and allowed a linear end of the broken end of 0.2 mm. This not only increases the formation of callus in indirect healing but also reduces the negative effects of stress occlusion.

An LLP alone not only protects the blood supply of the non-union site but also provides strong tension side stability for the femur. The LLP is different from the traditional screw-plate structure which depends on the interface of the bone and plate [42], in that its fixed angle structure prevents contact between the plate and the bone surface [43, 44]. Furthermore, an LCP can reduce the damage to the soft tissue with the aid of an insertion guide [45]. In addition, exposure of the non-union, cleaning of the dead bone, and removal of fibrous scar tissue provide a great osteogenic environment for subsequent bone grafting. Finally, the bicortical stability of the medial pressure of the J-bone limits micromotion, which creates an excellent mechanical environment for the repair of the non-union, thus promoting the indirect healing of the non-union. In addition, the J-bone is the gold standard for the treatment of non-union because of its complete histocompatibility, strong osteoinduction, osteoconduction, and osteogenic activities [46]. It is important for the J-bone to provide direct structural continuity at the site of the non-union, making up for the defect of the cancellous bone graft [47]. The medial protective plate maintains the filling and support of the J-bone so that the medial protective plate and J-bone can increase the medial compressive and bending strength and provide a reliable initial mechanical environment for local fibrous cartilage calcification and preliminary connection of the epiphysis. The medial protective plate and bicortical J-bone can also prevent the failure of the lateral plate internal fixation after surgery so that patients can recover and exercise early without delay, which is conducive to the functional recovery of the knee joints and osteophyte healing.

This study had several limitations, including clinical evidence from this study was a retrospective study, and large samples, randomized, and controlled clinical studies should be used to provide evidence. In the finite element analysis, we simplified the model without considering the effects of the actual femoral ligaments and joint capsules. But these results are enough to show the effectiveness of DP+J-bone.

In summary, a double steel plate combined with sacrum bone (J-bone) for the treatment of non-union of the distal femur with a bone defect has the advantages of less surgical trauma, early rehabilitation exercise after surgery, a high bone healing rate, and a satisfactory rate of functional recovery. Therefore, the DP+J-bone technique may be another technique for the successful treatment of ANFBD (especially in those with severe posterior medial defect).

## Data Availability

Please contact author for data requests.

## Abbreviations

ADFNBD: Atrophic Distal Femur Non-Union With Bone Defect
J-bone: J-shaped iliac crest bone graft
DP: Double-plate
MP: Medial plate
LLP: Lateral locking plate
LLP-only: LLP only group
DP-only: DP only group
DP+J-bone: DP with a J-bone group
LLP+J-bone: LLP with a J-bone group
LCP: Locking compression plate
3D: Three-dimensional
CT: Computed tomography
DICOM: Digital imaging and communications in medicine
LISS: Less invasive stabilization system
FEA: Finite element analysis
